# Fast Tracking—Vaccine Safety, Efficacy, and Lessons Learned: A Narrative Review

**DOI:** 10.3390/vaccines10081256

**Published:** 2022-08-04

**Authors:** Jason C. Wong, Crystal T. Lao, Melanie M. Yousif, Jacqueline M. Luga

**Affiliations:** College of Pharmacy, Western University of Health Sciences, 309 E. Second St., Pomona, CA 91766, USA

**Keywords:** Fast Track, vaccine, safety, efficacy, lessons, review, pandemic, FDA, COVID-19

## Abstract

(1) Background: The COVID-19 pandemic has led to the fast-tracked development of vaccines under emergency use authorization. In light of the growing concerns about fast-tracked vaccines, this article reviews the safety, efficacy, and lessons learned of previously fast-tracked vaccines. (2) Methods: An article search regarding the safety and efficacy of fast-tracked vaccines was done in PubMed, Embase, Web of Science, and ScienceDirect. Of the 104 results, 24 articles were included. Five articles about BiovaxID, THERATOPE^®^, Sipuleucel-T, and AIDSVAX were also reviewed. (3) Results: The overall efficacy was shown to be 77–100%, with seroprotection against the viruses ranging from 87 to 100%. The antibody responses for optimal protection against the viruses fall within 85–97%. Generally, the fast-tracked vaccines were well-tolerated and had few significant adverse events, except for the H1N1 pandemic vaccine and its association with narcolepsy. To have accurate, precise, and timely fast-tracked vaccines, communication, sharing resources/data, and improving the current structures/outbreak operations are crucial. (4) Conclusions: This review found the FDA’s fast-tracking process for vaccines to have rigorous standards similar to the normal process. The previous fast-tracked vaccines were safe and efficacious. The lessons drawn from previous studies highlighted the significance of planning and utilizing global resources during significant outbreaks.

## 1. Introduction

With the rising concerns regarding fast-tracked vaccines in society throughout this unprecedented pandemic, it is crucial to review the current literature to assess the safety and efficacy of fast-tracked vaccines from the past. Each time humanity has suffered a life-threatening pandemic, lessons were learned and are highlighted in this review. Vaccine fast-tracking has been a long-standing practice to meet emergent medical needs. However, little has been studied about the overall safety, efficacy, and lessons learned.

**Figure 1:**
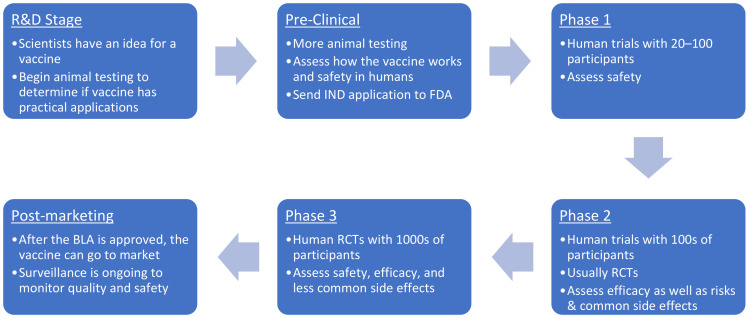
**The FDA’s standard vaccine approval process.** Abbreviations: R&D = Research & Development; IND = Investigational New Drug; FDA = Food and Drug Administration; RCT = Randomized Controlled Trial; BLA = Biologics License Application. After receiving the IND application, the FDA assesses the safety, preclinical data, and laboratory tests to ensure the tests were conducted according to Good Laboratory Practices. Once the IND application has been approved, human trials can begin. In addition to clinical data, the FDA also evaluates the manufacturing process and facilities for regulatory compliance. Manufacturers must show consistency among lots, specifically for the identity, sterility, purity, and potency standards. After a consistent manufacturing process has been developed and the clinical trials have been completed, a BLA is sent to the FDA. While a team of diverse scientific experts evaluates the BLA, the FDA also analyzes the risks and benefits of the vaccine for the population that will receive it. Once the BLA is approved, the vaccine can be distributed in the United States, and post-marketing surveillance is conducted to further ensure safety.

The fast-track approval process has been in the spotlight due to COVID-19, raising some public concerns about the efficacy and safety of fast-tracked vaccines and the vaccine approval process. To understand the fast-tracking process, the standard approval process for vaccines must first be clarified (refer to Figure 1).

The U.S. Food and Drug Administration’s (FDA) Center for Biologics Evaluation and Research (CBER) oversees vaccine approval. In the Research and Discovery (R&D) stage, scientists conduct animal testing to determine if the vaccine has practical applications. Then, in the Pre-Clinical Phase, they determine the safety and effectiveness in animals. Once the Investigational New Drug (IND) application has been submitted and approved, the Clinical Development stage for human trials is started. There are three phases, which usually progress in a sequence but may overlap. Phase 1 trials assess their safety in a small group. Phase 2 trials are conducted to determine their efficacy in a larger group. Finally, in phase 3 trials, the vaccine’s safety and efficacy are assessed in thousands of people [1,2].

After a reliable and consistent manufacturing process has been developed and the clinical trials have concluded, the manufacturer will submit a Biologics License Application (BLA) [1,2]. Once the BLA is approved, the vaccine may be distributed and marketed in the United States. Potential adverse effects are then monitored with post-marketing surveillance systems, such as the Vaccine Adverse Event Reporting System (VAERS) and the Centers for Disease Control and Prevention’s (CDC) Vaccine Safety Datalink (VSD) [1,2,3]. The complete process typically spans several years.

**Figure 2:**
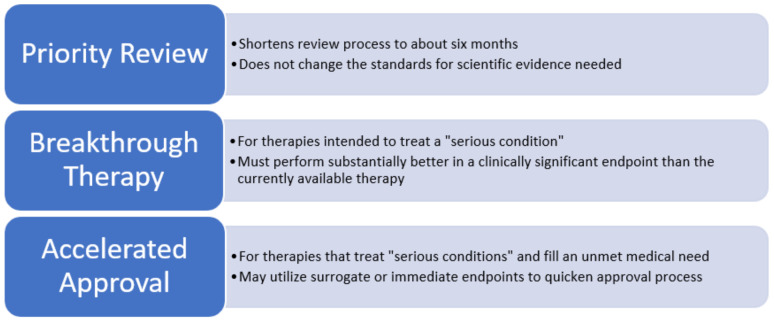
**FDA’s Rapid Approval Processes of Priority Review, Breakthrough Therapy, and Accelerated Approval.** A “serious condition” requires consideration of how the drug will affect the condition’s progression, survival, and daily functioning. A clinically significant endpoint measures an effect on irreversible morbidity or mortality (IMM) or on signs/symptoms that represent severe consequences of the condition. For a drug to “fill an unmet medical need”, it must either be potentially better than the current therapy or provide a new treatment where there was none previously. A Breakthrough Therapy designation must be requested, but, if a company has not requested a Breakthrough Therapy designation, the FDA may suggest that they submit a request if: (1) the FDA considers the drug meets the Breakthrough Therapy criteria after reviewing all the submitted data, and (2) the designation can help benefit the remaining drug development program. For Accelerated Approval, researchers use a clinically significant surrogate or intermediate endpoint, which can help save time in the drug approval process. However, the FDA must evaluate these endpoints to determine if they are scientifically acceptable. For Accelerated Approval, phase 4 trials are still conducted to confirm the clinical benefit. If a clinical benefit is confirmed, then the FDA will usually terminate the requirement. If the trials fail to confirm any benefit or do not demonstrate enough benefits to justify the drug’s risks, the FDA may withdraw approval for the drug.

**Figure 3:**
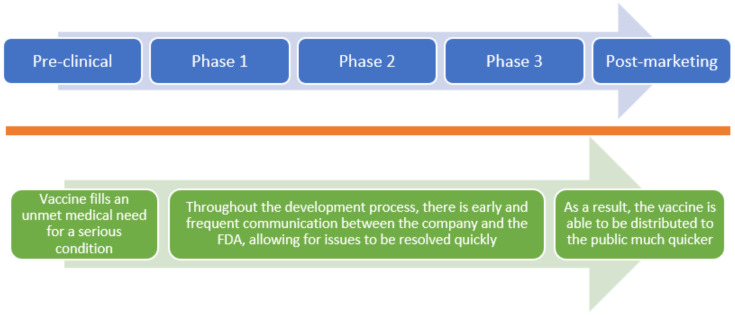
**FDA’s Fast-Track approval process.** The figure above shows the standard approval process in blue, while the Fast-Track approval process is shown in green. After the drug company requests Fast Track, the FDA will evaluate the drug and make a decision within 60 days. With a Fast Track designation, the sponsors can also request a Rolling Review, so the manufacturer can submit the finished BLA or NDA to the FDA for review instead of waiting for the entire application to be completed. Additionally, therapeutic candidates that have been permitted a Fast Track designation are also eligible for Accelerated Approval and Priority Review.

The FDA has four distinct programs for hastening vaccine development and review: Accelerated Approval, Breakthrough Therapy, Priority Review, and Fast Track (refer to Figure 2 and Figure 3) [4]. These programs may be used for vaccines and medications.

Before approval, the drug must undergo a detailed review process, including a Standard Review or Priority Review. The Standard Review usually spans ten months, whereas the Priority Review can shorten the process to about six months. The Priority Review does not change the length of the clinical trials or the scientific standards for the evidence needed [5].

Only therapies designed to treat a serious condition may be granted a Breakthrough Therapy designation. The preliminary clinical data must show that it performs substantially better in a clinically significant endpoint than the currently available therapy [6]. A Breakthrough Therapy designation must be requested. However, if a manufacturer has not sought out a Breakthrough Therapy designation, the FDA may advise that they submit a request if the therapy meets certain criteria (refer to Figure 2 for more detail) [6].

Accelerated Approval also hastens the development and increases the availability of therapies for serious conditions to fill an unmet need. For Accelerated Approval, researchers may use an FDA-approved clinically significant surrogate or intermediate endpoint to help save time in the drug approval process. Furthermore, the drug company will still need to conduct phase 4 trials to confirm the clinical benefits [7].

The focus of the narrative review is the Fast Track designation (see Figure 3). To qualify, the therapy must treat serious conditions and fill an unmet medical need. At any time during the development process, the drug company must request a Fast Track designation. Within 60 days, the request will be evaluated for a decision by the FDA. If the request is approved, constant correspondence between the FDA and the drug company is recommended so that any issues can be quickly resolved [8]. This narrative review aims to evaluate and discuss the literature regarding the efficacy and safety of fast-tracked vaccines and the lessons learned from launching fast-tracked vaccines.

## 2. Materials and Methods

**Figure 4:**
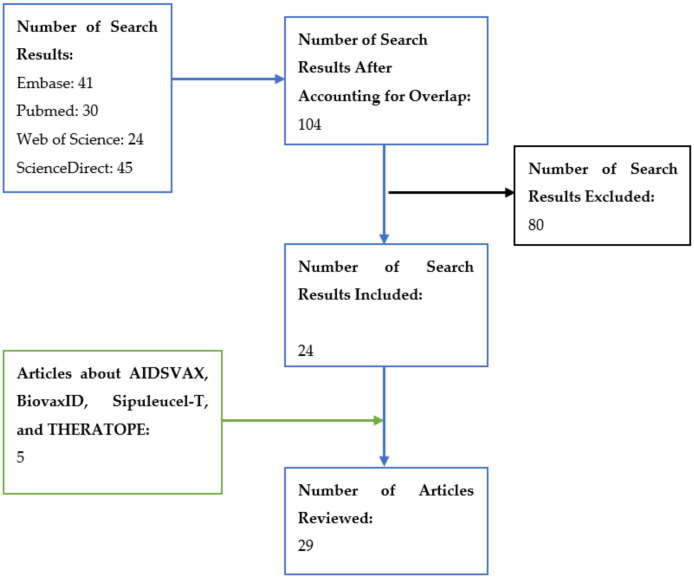
**Literature search and article inclusion/exclusion.** For Embase, PubMed, and Web of Science, the search terms used were “vaccine”, “fast track”, and “safety”, without any filters. For ScienceDirect, additional search terms “human”, “efficacy”, and “immunization” were used, as well as the “Research Articles” filter.

This narrative review received an exempt status from the Institutional Review Office of Western University of Health Sciences. A literature search was conducted in March 2021 to obtain articles that pertained to the efficacy and safety of fast-tracked vaccines. No filter was placed on the publication date during the search. When searching in PubMed, Embase, and Web of Science, we used the keywords “vaccine”, “fast track”, and “safety”, without any filters. An adjusted literature search was conducted in ScienceDirect by adding the terms “human”, “efficacy”, and “immunization” to the previous keywords, as well as applying the “Research Articles” filter due to ScienceDirect, as it is such a sensitive article search system (see Figure 4).

The PubMed search generated 30 results, while Web of Science had 24 results. Embase had 43 results, but two articles appeared twice on the results page, so the actual number was 41 articles. After modifying the initial search parameters, ScienceDirect yielded 45 results. After accounting for the overlap of articles between databases in the literature search, 104 articles were found in the literature search.

Among the results, eight randomized clinical trials (RCTs) were critically appraised by two independent individuals critically appraised. The RCTs were also assessed with the Cochrane Risk of Bias (RoB) Tool (Table 1), which was designed for randomized studies. After the evaluation, four clinical trials were determined to have an acceptable risk of bias and were included in the narrative review. Although Mire et al., 2015 were determined to have low risk of bias, it was excluded from the review due to the patient population being nonhuman primates, limiting the external validity of the results. Additionally, Sirima et al., 2017 was determined to have a primarily low risk of bias, but there were some concerns for bias due to the small sample size (European *n* = 30, African *n* = 36). However, it was included in the review due to addressing both vaccine safety and efficacy in a human study population and having a high internal validity from randomization.

There is no standardized method for evaluating nonrandomized studies. The Cochrane Risk of Bias in Non-Randomized Studies—of Interventions (ROBINS-I) tool and the Newcastle–Ottawa Scale (NOS) were developed to assess nonrandomized studies in the context of systematic reviews and meta-analyses, respectively [9,10]. In addition, the risk of bias assessment for a nonrandomized study should address the preintervention, at-intervention, and post-intervention features. However, some of the nonrandomized studies included in this review did not address all three features. As such, these methods are not appropriate for assessing the non-RCTs included in this narrative review. Therefore, the non-RCTs included in this narrative review were not able to be assessed for the risk of bias. Three authors assessed the remaining articles for their relevance to the topic of safety and efficacy of fast-tracked vaccines. The inclusion criteria for this narrative review were articles that discuss the safety, efficacy, and/or lessons learned of previously fast-tracked vaccines. Including the previously mentioned clinical trials, 80 articles were excluded for the following reasons: they did not pertain to the topic of fast-tracked vaccines, the vaccines were under the purview of regulations of countries outside of the United States, making it difficult to draw conclusions due to different standards, the vaccine in question was hypothetical and had not yet been created, they had incredibly sparse information, they were only tangentially related to the topic of the review, and the full text was unable to be obtained despite using multiple databases.

From the literature search, 26 articles were determined to be pertinent and appropriate to the topic of this narrative review. Some articles discussed specific vaccines (i.e., BiovaxID, THERATOPE^®^, Sipuleucel-T, and AIDSVAX), and a separate search was conducted for articles that presented the conclusions for the development of those vaccines, yielding 5 articles. In total, 29 articles were included in this narrative review (see Figure 4).

This narrative review fulfilled the criteria in the International Narrative Systematic Assessment (INSA) tool necessary for a high-quality review. The criteria were: (1) background of the study clearly explained; (2) objective was clear; (3) description/motivation of the selection of the studies; (4) description of the characteristics of the included studies was clear in the paper; (5) presentation of the results (paragraphs, tables, and synthesizing of data); (6) the conclusion was clear; and (7) the author(s) declare(d) that there were no conflicts of interest regarding the publication of the article [11].

## 3. Results

### 3.1. Recent History and Previous Fast-Tracked Vaccines

The timely development and approval of vaccines are essential to the preventive aspect of the public health, especially during times of crisis and when responding to emerging (or reemerging) infectious diseases. Søborg et al., 2009 discussed the use of clear communication between governments and the vaccine industry and the use of incentives to facilitate the timely development of vaccines and novel technologies [12]. History has shown that rapid development and implementation are possible when both the government and the public are committed to the cause. In the case of the polio vaccine, it only took one year for the vaccine to go through large-scale double-blinded, placebo-controlled studies, demonstrate its safety and efficacy, and advance to licensure. Søborg et al., also proposed methods to improve the current fast-tracking process. These include having an established framework for carrying out fast-tracked clinical trials, increased communication between vaccine developers and regulatory bodies, a process for approving with the understanding that required paperwork can be submitted retrospectively, better reception of the surrogate endpoint data, and more flexibility regarding the composition of vaccines [12].

Muzumdar and Cline 2009 delved into the supply and demand issues, as well as the policy options associated with the vaccine industry [13]. Their analysis found that the research and development of vaccines could cost approximately USD 800 million and take at least ten years to complete, partially due to the strict manufacturing regulations. These high development costs are only one of the barriers that limit the number of vaccine manufacturers. “Push” policies tackle the supply side and attempt to assist vaccine developers by easing the burden of development costs. One of the “push” strategies that they discussed was the FDA’s fast-tracking process, highlighting BiovaxID as an example. On the other hand, “pull” strategies focus on the demand side and increase the reception of immunizations [13]. The push–pull policies are crucial to supply chain management and improve vaccine development by increasing the production of and access to vaccines.

In a 2016 perspective article spurred by the then-current outbreak in North and South America, Thomas et al., discussed the possibility of fast-tracking the Zika virus vaccine development [14]. There were two DNA ZIKA vaccine candidates at the time of publication, and they had only entered phase 1 human safety trials. For any emergency, demonstrating a vaccine’s clinical efficacy and safety is key. Furthermore, to accelerate the production process of a Zika vaccine, some development activities and clinical evaluations will need to be conducted simultaneously. It is also crucial to remember the realities of scientific research [14]. Vaccines that are granted a Fast Track designation still need to go through trials to demonstrate their safety and efficacy.

In more recent events, the FDA’s fast-tracking of vaccines has been highlighted during the COVID-19 pandemic. In their 2020 commentary article, Limaye, Sauer, and Truelove asserted their support of the FDA’s fast-tracking process, but they also voiced concerns about how political motivations should not influence the scientific method [15]. There has always been a baseline level of vaccine hesitancy, but the addition of perceived political pressure and a “rushed” development of a vaccine may serve to decrease vaccine acceptance. As such, Limaye, Sauer, and Truelove stressed that, while fast-tracking is crucial during public health emergencies, innovative approaches are necessary to hasten the development time while maintaining the safety and efficacy standards. These approaches include the enhanced surveillance of adverse events and decision-making related to clinical trials being overseen by impartial advisory boards. Furthermore, Rappuoli et al., 2021, described the many changes in the vaccine technology brought about by COVID-19 [16]. One category of vaccines experiencing accelerated platform development is the synthetic RNA vaccines, which use synthetic genes cloned into a plasmid vector as a template for RNA vaccine synthesis. Since these vaccines are wholly synthetic and do not need a biological phase, they could quickly advance between clinical phase trials. Although manufacturing RNA vaccines is simpler than conventional vaccines, before the COVID-19 pandemic, the scale was never large enough to warrant clinical trials [16]. However, the pandemic has brought about an urgent need for vaccines and has presented an opportunity for the fast-tracked development of many types of vaccines.

### 3.2. Efficacy

Efficacy of vaccines from the included articles is summarized in Table 2.

### 3.3. Safety

Safety of vaccines from the included articles is summarized in Table 3.

### 3.4. Lessons Learned

Lessons learned from vaccines in the included articles is summarized in Table 4.

## 4. Discussion

### 4.1. Efficacy

The efficacy of previous fast-tracked vaccines was presented in this review based on the markers determined by the individual studies (refer to Table 2). Steiner-Monard et al., 2009 evaluated the P27A peptide vaccine in malaria-exposed African adults and malaria-nonexposed Europeans in a fast-track randomized trial in Switzerland [17]. The sample included 40 malaria-exposed and 16 malaria-nonexposed subjects. Patients were administered the P27A antigen IM adjuvanted with glucopyranosyl lipid adjuvant stable emulsion (GLA-SE), Alhydrogel, or the control rabies vaccine (Verorab). Although the study did not report on efficacy, the results reported immunogenicity, which can help assess the efficacy. There was a specific humoral immune response represented by the mixed cellular immunity from Th1 and Th2 and the induced IgG antibody response by p27A that was able to inhibit parasite growth [17].

Sirima et al., 2017 focused on the immunogenicity of the recombinant Plasmodium falciparum Apical Membrane Antigen 1 Diversity Covering (AMA1-DiCo) malaria vaccine adjuvanted with either GLA-SE or Alhydrogel in a randomized, double-blinded trial with European and African adults [18]. The primary immunogenic response noted was an increase in IgG. The AMA1-DiCo malaria vaccine with Alhydrogel group caused a 100-fold IgG increase from the baseline and a 200–300-fold IgG increase when adjuvanted with GLA-SE. After immunization, the African volunteers’ increase in IgG levels was four times greater than that of European volunteers. An IL-5 ELISPOT assay was used to display the robust Th2 cell response in more than 50% of the volunteers [18].

De Whalley and Pollard 2013 reviewed the 2009 influenza A (H1N1) vaccination in children in the UK [19]. The AS03-adjuvanted vaccine was shown to effectively prevent H1N1 influenza infection starting seven days after vaccination. In England, the AS03-adjuvanted vaccine had 77% effectiveness in children under ten years old and 100% in people aged 10–24 years. This article also discussed a study in Sweden conducted in 2009, which reported that the AS03-adjuvanted vaccine was highly effective at 89–92% in children and 69–89% in adults. Overall, this UK study proved fast-tracked vaccines to have impressive efficacy [19].

McVernon and Nolan 2011 reviewed Panvax^®^, a monovalent inactivated adjuvanted vaccine designed for the 2009 influenza (H1N1) pandemic [20]. Their review primarily reported immunogenicity, which showed an optimal antibody response and >90% seroprotection for ages 6 months to 64 years old after one 15-μg dose of Panvax^®^ in the accelerated clinical trials, allowing the Panvax^®^ vaccine to achieve the necessary criteria for licensure [20]. According to McVernon and Nolan 2011, the CDC had conducted a case–control study that found effectiveness of monovalent vaccines, including Panvax^®^, to be 62% (95% CI: 30–79%) across all age groups.

Cox and Hollister 2009 reviewed the efficacy of the FluBlok vaccine based on four studies: PSC01, PSC03, PSC04, and PSC06. [22]. In PSC01, adults aged 18–49 years were randomized to receive either FluBlok dose of 135 μg (*n* = 153) or 75 μg (*n* = 151) or saline placebo (*n* = 154). PSC04 also compared FluBlok to a placebo (*n* = 2304) in the same age group but only examined a 135-μg dose (*n* = 2344). Older patients were included in PSC06 (age 50–64 years) and PSC03 (age ≥ 65 years), which compared FluBlok 135 μg (PSC03: *n* = 436; PSC06: *n* = 300) to trivalent Fluzone^®^ (PSC03: *n* = 433; PSC06: *n* = 302). In all four studies, the serum antibody responses were evaluated 28 days following the vaccination. Adults ≥65 years old showed 95% seroprotection against A/H1N1 (95% CI 92–97) and 97% against A/H3N2 (95% CI 94–98) with the 135-μg dose. Similarly, a robust efficacy was seen in adults aged 50–64 years, with 96% seroprotection against A/H1N1 (95% CI 94–98) and 85% against A/H3N2 (95% CI 81–89). For adults aged 18–49 years in PSC01, 135 μg of FluBlok provided 87% seroprotection against A/H1N1 (95% CI 81–92) and 100% against A/H3N2 (95% CI 98–100), while PSC04 showed 98% against A/H1N1 (95% CI 97–99) and 96% against A/H3N2 (95% CI 94–98) [22]. In 2011, Cox and Hashimoto reviewed the efficacy of the FluBlok vaccine vs. placebo based on the same four studies [21]. Based on PSC01 and PSC04, they determined that FluBlok had an efficacy of 85.5% overall against culture-confirmed CDC-ILI, an influenza-like illness specified by the CDC. In addition, Fisher’s exact test was used to conduct a post hoc analysis, which showed a statistically significant reduction in culture-confirmed CDC-ILI in the FluBlok group vs. the placebo (*p* = 0.0146) [21].

Ball’s 2009 article discussed the immunogenic response to the H1N1 vaccine without specifying the efficacy. Researchers examined blood samples of 120 adults 21 days after receiving a 15-μg injection of the H1N1 vaccine. The results showed approximately 97% of these adults had enough antibodies for optimal protection against the virus in 97% (116/120) of the adults who received the 15-μg dose, shown by antibody titers of at least 1:40 [23,24].

In Reinis 2008, the immunogenic abilities of BiovaxID were assessed [25]. BiovaxID is a therapeutic vaccine targeted towards non-Hodgkin’s lymphoma. Phases I and II of this vaccine’s trials were assessed by this article, but phase III was still in process at the time of publication. The phase I trial of BiovaxID included five subcutaneous injections of BiovaxID to 41 patients with follicular non-Hodgkin’s lymphoma in primary or secondary remission after chemotherapy treatment. Phase I showed 85% of the patients had antibody responses, while 35% had cellular responses. In addition, 87% of the patients who responded to the vaccine were able to remain tumor-free. Phase I also revealed a progression-free period for vaccinated patients with a response of 7.9 years compared to those who did not respond to the vaccine, with only a progression-free period of 1.3 years. Phase II of the clinical trial included four monthly, subcutaneous injections of BiovaxID with a sample size of 20 in complete remission after six or more monthly cycles of proMACE chemotherapy. The follow-up data for phase II of this trial showed 45% disease-free survival and a 95% overall survival rate among patients. Phase III of this trial took place at the time of this article and consisted of a randomized, double-blinded clinical trial where 629 patients received five vaccine doses. After vaccination, disease-free survival increased by 60% in both the control and BiovaxID groups by 21.2 and 33.8 months, respectively (*p* = 0.047). It also revealed BiovaxID treatment after 36 months showed approximately 100% improvement in the median disease-free survival (*p* = 0.024) [25].

Another novel cancer vaccine is the THERATOPE^®^ vaccine, which was granted FDA Fast Track status in May 2000 as an adjunct to chemotherapy in metastatic breast cancer treatment. In one study, 40 patients with ovarian and breast cancer were each given five doses of the vaccine following high-dose chemotherapy/autologous stem cell transplant (HDC/ASCT). The results indicated that the chances of death and relapse were 2-times and 1.7-times higher, respectively, for patients who did not receive the vaccine [26]. In the pivotal phase III trial, 1030 women with metastatic breast cancer and who received cyclophosphamide were recruited. Antibody testing was conducted at week 12 and showed high specific immunoglobulin G titers in the treatment group, but there were no detectable antibodies in the control group. However, the median survival time between the treatment and control groups was not statistically significant (23.1 months vs. 22.3 months) [27]. Therefore, the development of THERATOPE^®^ was hampered as researchers tried to ascertain why the clinically significant antibody titers were not translating into an increased survival.

Pérez-Vargas et al., 2006 focused on the efficacy of the Rotarix vaccine in Latin America [28]. Given that Rotarix was based primarily on an attenuated serotype G1 rotavirus strain, the authors noted efficacy issues of Rotarix against the vast range of virus strains. The efficacy of the vaccine was 85% against severe rotavirus gastroenteritis and related hospitalization (*p* < 0.001 vs. the placebo). In more severe gastroenteritis cases, the efficacy amounted to 100%. It also reduced the hospitalizations of diarrhea by 42% (95% CI). The overall efficacy of Rotarix against the G1 rotavirus strain was seen to be 91% [28].

### 4.2. Safety

Safety is also paramount when establishing effective therapeutic agents (refer to Table 3). As previously mentioned, Steiner-Monard et al., 2019 evaluated the P27A peptide vaccine for malaria. In phase 1a of the trial, the most common systemic adverse effects were tiredness (48.5%) and headache (29.2%). There were no significant abnormal vital signs noted or other severe effects. In phase 1b, 64.1% experienced adverse effects of fatigue and headache. Similar to phase 1a, no significant abnormal vital sign measures were observed following injection [17].

Sirima et al., 2017 scrutinized the recombinant Plasmodium falciparum AMA1-DiCo malaria vaccine adjuvanted with GLA-SE or Alhydrogel in European and African adults after a randomized, double-blinded trial. For cohort A (France), 1 out of 30 participants experienced epilepsy unrelated to the vaccination. There were no further systemic reactions reported. However, many local injection site reactions were seen in both the Alhydrogel and GLA-SE groups (83% vs. 100%), and the reactions resolved without recurrence. For cohort B (Burkina Faso), local reactions were experienced by both the GLA-SE and placebo groups after the first injection (33% vs. 17%). Systemic reactions were seen in 22% of the GLA-SE group and 22% in the placebo group. In the placebo group, urticaria was reported after both immunizations and was unrelated to the vaccine [18].

Pérez-Vargas et al., 2006 reviewed the safety profiles of Rotarix and RotaShield. RotaShield was removed from the US market in 1999 due to its estimated risk of developing intussusception in between 1 in 10,000 and 1 in 32,000 vaccine receivers. Rotarix was later developed, but the preliminary clinical data on Rotarix was not sufficient to make conclusions about its safety [28].

As previously mentioned, the THERATOPE^®^ vaccine was granted FDA Fast Track status in May 2000 for its indication as an adjunct to chemotherapy in metastatic breast cancer treatment. In the 2003 R&D profile article, preliminary results from a then-ongoing phase II trial in the treatment of ovarian cancer indicated that the vaccine was well-tolerated with mild flu-like symptoms [26]. The phase III trial found that THERATOPE^®^ was well-tolerated, with injection site reaction and nausea/vomiting as the most common adverse reactions. However, there was no overall benefit in progression or survival in women with metastatic breast cancer, and the development of THERATOPE^®^ was stalled [27].

Another innovative vaccine is Sipuleucel-T, otherwise known as APC 8015 or Provenge ^®^, that was granted Fast Track status in November 2005 for its vaccine indication of asymptomatic, metastatic, androgen-independent prostate cancer. According to the 2006 R&D profile article, data from two phase III trials, D9902A and D9901, showed that Sipuleucel-T was well-tolerated, with mild fever and chills being the most common adverse events in trial D9902A [29]. Higano et al., 2009 provided an integrated analysis of the D9901 and D9902A trials, which concluded that a survival benefit was conferred to patients who received Sipuleucel-T compared to patients who received a placebo [30]. Additionally, Sipuleucel-T was associated with a modest adverse effect profile. The most common adverse effects were pyrexia, headache, asthenia, chills, dyspnea, vomiting, and tremors, usually grades 1 or 2 in severity and 1 to 2 days in duration [29,30].

As described by Kantoff et al., 2010, a subsequent double-blinded, placebo-controlled, multicenter phase 3 trial was conducted in patients with metastatic castration-resistant prostate cancer and randomly assigned to receive either Sipuleucel-T (*n* = 341) or a placebo (*n* = 171) [31]. Although the time to disease progression was not significantly affected, Sipuleucel-T was found to increase the overall survival. Compared to the placebo group, the most common adverse events seen in the Sipuleucel-T group were influenza-like illness, chills, fever, headache, hyperhidrosis, myalgia, hypertension, and groin pain. Furthermore, only three patients in the Sipuleucel-T group experienced infusion-related adverse effects that led to study withdrawal. The researchers concluded that the adverse events seen more often in the Sipuleucel-T group were consistent with cytokine release and that Sipuleucel-T was well-tolerated while prolonging survival in the study patients [31].

Similar to the current COVID-19 pandemic, there was an urgent need for vaccines in the 2009 influenza A H1N1 pandemic. In their 2014 review article, Barker and Snape focused on the association between the H1N1-AS03-P adjuvanted pandemic vaccine and an apparent increase in the incidence of narcolepsy [32]. Adolescents were seen to develop narcolepsy following the H1N1-AS03-P vaccine, which prompted investigations by the European Centre for Disease Prevention and Control (ECDC) and the Vaccine Adverse Event Surveillance and Communication Consortium (VAESCO) in late 2010. The results of these investigations, along with the studies conducted in Finland, Sweden, Ireland, and the Netherlands, supported an increased risk of post-vaccination narcolepsy. Although the researchers concluded that diagnostic bias and media coverage might have influenced events, the increased risk was still deemed significant [32]. Although the underlying mechanism of post-vaccination narcolepsy remains largely unknown, Barker and Snape underscored the importance of alert clinicians to adverse events reporting, showing how crucial international cooperation is in post-marketing surveillance strategies for future mass vaccination campaigns [32].

Wijnans et al., 2011 analyzed the safety profile of pandemic H1N1 vaccines in children and adolescents [33]. The 25 clinical studies included 10,505 subjects between 6 months and 23 years, ranging from healthy individuals to those with underlying medical conditions. Wijnans et al., examined case reports involving the monitoring of children and adolescents sent to the World Health Organization (WHO) during the pandemic, but there were no vaccine-related adverse events reported in the monovalent nonadjuvanted inactivated or live-attenuated pandemic H1N1 vaccines. Wijnans et al., reviewed two studies regarding MF59-adjuvanted pandemic vaccines and five studies for the AS03-adjuvanted pandemic vaccines. The studies concluded that the adjuvanted vaccines were more reactogenic compared to nonadjuvanted vaccines and were generally well-tolerated in terms of adverse events [33].

### 4.3. Lessons Learned

Fast-tracking vaccines require speed, accuracy, and precision without compromising the integrity of the vaccine. As emergencies continue to require fast-tracked vaccines, it is important to meet the public’s concerns regarding safety and efficacy with scientific data. Over the years, many vaccines have been fast-tracked around the world. By studying the lessons learned from these fast-tracked vaccines, the knowledge can be used to improve the fast-tracking process (refer to Table 4).

A 2003 R&D profile presented information about VaxGen’s AIDSVAX B/B and AIDSVAX B/E vaccines. Although these vaccines were granted the FDA Fast Track designation in 2002, the AIDSVAX B/B vaccine did not show statistically significant efficacy in Europe and North America phase III trials [34]. As reported by the AIDS Vaccine Advocacy Coalition (AVAC) in 2003, AIDSVAX did not prevent HIV infection in the study population. Although AIDSVAX did not enter the market, the clinical trials provided valuable data for the development of future HIV/AIDS vaccines [35].

Another vaccine of note is GlaxoSmithKline’s (GSK) Rotarix, a human-attenuated rotavirus vaccine. As discussed in Pérez-Vargas et al., 2006, Rotarix was licensed for use in Mexico, Kuwait, and the Dominican Republic [28]. However, approval from the FDA and the European Agency for the Evaluation of Medical Products was still pending. It was being evaluated in phase III clinical trials in Europe, Latin America, and Asia [28]. In countries like Mexico, the fast approval of the Rotarix vaccine could save thousands of lives. In contrast, the formal approval and introduction process could take years before it would be available to the public.

Then, during the 2009 influenza pandemic, many research and studies were conducted and critiqued. The first study of note is McVernon and Nolan 2009, which reviewed Panvax^®^, a pandemic influenza A (H1N1) vaccine. If the rapid containment of emerging viruses is to be achieved, they concluded that future pandemic vaccines should be developed to prevent infections of both pandemic and seasonal strains [20]. Another study, Wong et al., 2009, examined the antiviral role of Toll-like receptor-3 (TLR-3) agonists against avian and seasonal influenza viruses and found that preclinical studies support the potential of TLR-3 agonists for prophylaxis against influenza. The study confirmed that regulatory agencies should consider the fast track development of drugs for prophylaxis and the potential treatment of H5N1 [36]. Furthermore, Wijnans et al., 2011 emphasized the importance of improving the surveillance methods and infrastructure, as well as a multinational collaboration, in conducting vaccine safety studies [33]. The last study to focus on the 2009 influenza pandemic, de Whalley and Pollard 2013, reviewed two monovalent vaccines in the UK [19]. They found that using vaccines with oil-in-water adjuvant systems resulted in great immunogenicity in the younger population. They also highlighted the importance of substantial developments in understanding vaccinology, influenza epidemiology, and pandemic policy planning [19].

Many serious infectious diseases remain for which a fast-tracked vaccine may hold incredible life-saving potential. For example, regarding the 2015 Ebola outbreak in West Africa, Lai et al., reported using a recombinant vesicular stomatitis virus-based Ebola vaccine (VSVΔGZEBOV) in a physician who had a needlestick in an Ebola treatment unit. Although it is uncertain whether the vaccine influenced the physician’s survival, Geisbert’s editorial about this incident emphasized the importance of having a sufficient supply of safe and effective vaccines that can be rapidly deployed in emergency situations [37]. Additionally, during the Ebola outbreak, clinical trials, including the EBOVAC-Salone study, were fast-tracked to discern possible vaccines and treatments. To expedite timely and appropriate research projects, the experiences suggest that research should be more intimately integrated into outbreak response planning [38].

Feldmann, Feldmann, and Marzi 2018 also stressed the importance of having resources prepared in advance to effectively respond to the Ebola virus (EBOV) in the future and prevent poor immunogenicity. Feldmann, Feldmann, and Marzi suggested studying other EBOV antigens, so that time, money, and better results can be achieved earlier rather than having extra expenses and reduced optimization. The epidemic demonstrated the lack of preparation and limitations in our public health response. Since then, better planning and preparation have been discussed and implemented [39]. Another study on the West African Ebola epidemic by Watle, Norheim, and Røttingen 2016 reiterated that good coordination and collaboration during future epidemics is necessary to develop a vaccine safely and effectively [40]. Additionally, it is important to improve WHO’s current structures for the response to and preparation for future epidemics. Other lessons about partnerships, ethical considerations, human resources, participant recruitment, operational issues, and post-outbreak transitions were also discussed in Mooney et al., 2018 [38].

Throughout the fast-tracked vaccines mentioned in this narrative review, the lessons learned can be summarized in the following points: the importance and benefits of speed, the use of multinational collaboration, flaws in pandemic policy planning, and the need to improve outdated surveillance and infrastructures. Many of the studies about the fast-tracked vaccines, such as Dhanda et al., 2020, stated that the post-marketing surveillance requires major revisions and updates in the systems to create active methods for reporting and organizing data in order to effectively and safely assess how these vaccines perform in real-life situations [41]. Learning from these lessons and discovering the system’s flaws can help improve the safety and efficacy of fast-tracked vaccines. Furthermore, the value of fast-tracked programs still remains, as they provide the prompt approval of products to treat serious, fatal conditions caused by viruses such as H1N1 and COVID-19.

### 4.4. In the Context of COVID-19

Since the COVID-19 pandemic started in March 2020, the scientific community has raced to develop a vaccine to help slow the relentless spread of the disease that has resulted in millions of deaths worldwide. In such an unprecedented situation, a regulatory process for the timely assessment of vaccine safety and efficacy was of the utmost importance [42]. The only COVID-19 vaccines currently available to the US public are Pfizer-BioNTech (BNT162b2 and COMIRNATY); Moderna (mRNA-1273, CX-024414, and SPIKEVAX); and Janssen. In comparison to the inactivated and live-attenuated vaccines, the nucleic acid-based technology of the Pfizer and Moderna vaccines was less familiar and raised safety concerns [43]. Specifically, Pfizer and Moderna are mRNA vaccines that have demonstrated the ability to enhance neutralizing antibodies.

Mass vaccination and regulatory harmony are essential to controlling the viral spread and preventing the rise of new variants. The current COVID-19 vaccines have demonstrated a reduction in mortality and severe infection [43,44]. The median review time for permitting Emergency Use Authorizations (EUAs) for COVID-19 vaccines was 21 days, much less than the median of 12 months based on 21 vaccines that the FDA approved between 2010 and 2020. In spite of the urgency of the pandemic situation, the Pfizer, Moderna, and Janssen vaccines still underwent clinical trials to receive approval from the FDA, European Medicines Agency (EMA), and Health Canada (HC). Additionally, these clinical trials were either randomized, double-blinded, had primary clinical endpoints, and/or used an active comparator [44]. In the interest of worldwide health, regulatory harmony and scientific integrity between nations are necessary to provide safe and effective vaccines promptly to all peoples.

A pharmacovigilance analysis used data on adverse events following immunization (AEFIs) sent to VigiBase from 1 January 2020 to 17 January 2021 to compare the safety of Pfizer and Moderna vaccines to influenza vaccines [45]. The analysis revealed that both vaccines had statistically significant associations with common AEFIs. Furthermore, COVID-19-vaccinated people were more likely to experience myalgia, headache, fatigue, and pyrexia, while influenza-vaccinated people were more likely to experience injection site reactions. In terms of more severe, rare adverse effects, the mRNA vaccines were shown to have a significantly higher risk for hypertensive crisis and supraventricular tachycardia, but they were also shown to have a lower risk of neurological complications compared to influenza vaccines [45]. Even though the process of fast-tracking and EUAs may seem reckless to some, expeditious review processes coupled with reliable scientific data have ensured that protective vaccines are available to the public. The experience of COVID-19 will hopefully leave scientists and regulatory authorities better able to streamline the process of delivering novel therapies to those in need.

### 4.5. Limitations

Narrative reviews have less-defined guidelines for conduct than systematic reviews and can be more at risk of bias. However, efforts were made to conduct a thorough literature search and include relevant articles. Additionally, although the literature search yielded few clinical trials, those articles were critically appraised to ensure the quality and acceptable risk of bias. Furthermore, we strived to discuss the articles unbiasedly, with our main purpose to clarify the FDA’s fast-tracking process and how it relates to the safety and efficacy of vaccines.

## 5. Conclusions

Although the FDA’s fast-tracking process was introduced by the FDA Modernization Act of 1997, it has since increased in importance and debate. Given the global COVID-19 pandemic, there has been a clear need for the timely development of safe and effective vaccines. The FDA’s fast-tracking process requires the careful deliberation of many details, such as regulatory/ethics review, trial design, safety and efficacy, and IND and BLA applications, as well as early and frequent communications with the FDA. Many past fast-tracked candidates were effective, but some were not approved due to safety issues. Many lessons emerged throughout the research process, such as clinical discoveries for H5N1 and the rotavirus, as well as the importance of planning, organizing, and combing resources during major outbreaks.

As more data become available, further research is needed to evaluate the safety and efficacy of fast-tracked vaccines. Future research may investigate vaccines and drugs with other rapid approval designations, such as Accelerated Approval, Breakthrough Therapy, Priority Review, and Emergency Use Authorization. Other avenues may explore specific vaccines, such as the COVID-19 vaccines. This narrative review focused on the fast-tracking process and its influence on vaccine safety and efficacy, as they are particularly relevant during the COVID-19 pandemic. However, it should be noted that the FDA has other fast approval processes available, which can be applied to vaccines, drugs, and other medical products. Furthermore, the experience gained from fast-tracked vaccines may serve as a valuable resource even for those vaccines going through the standard development process. The proper understanding and usage of these rapid approval processes can contribute to the timely development and increased public access of many medical breakthroughs.

## Figures and Tables

**Table 1:** RCTs and Risk of Bias using the Cochrane Risk of Bias Tool.

Article	Risk of Bias	Include or Exclude?
De Wit et al., 2015	Some concerns	Exclude
Langenberg et al., 2020	Some concerns	Exclude
Mire et al., 2015	Low risk of bias	Exclude
Steiner-Monard et al., 2019	Low risk of bias	Include
Higano et al., 2009	Low risk of bias	Include
Kantoff et al., 2010	Low risk of bias	Include
Sirima et al., 2017	Low risk of bias/Some concerns	Include
Gengenbacher et al., 2014	High risk of bias	Exclude

**Table 2:** Parameters and results of the studies reviewed for efficacy.

Publication Date	Study Name	Fast-Tracked Vaccine	Patient Population	Intervention	Results
2019	The Candidate Blood-stage Malaria Vaccine P27A Induces a Robust Humoral Response in a Fast Track to the Field Phase 1 Trial in Exposed and Nonexposed Volunteers [17]	P27A peptide vaccine against malaria	16 malaria non-exposed and 40 malaria-exposed subjects	P27A antigen IM adjuvanted with Alhydrogel, glucopyranosil lipid adjuvant stable emulsion, or control rabies vaccine (Verorab)	Specific humoral immune response represented by mixed Th1and Th2 cell mediated immunity as well as p27A-induced IgG antibody response able to inhibit parasite growth.
2017	Safety and immunogenicity of a recombinant *Plasmodium falciparum* AMA1-DiCo malaria vaccine adjuvanted with GLA-SE or Alhydrogel^®^ in European and African adults: A phase 1a/1b, randomized, double-blind multi-center trial [18]	Recombinant *Plasmodium falciparum* AMA1-DiCo malaria vaccine adjuvanted with GLA-SE or Alhydrogel^®^	Healthy European and African adults	European Adults: Intramuscular injection of AMA1-DiCo with either Alhydrogel^®^ (*n* = 15) or GLA-SE (*n* = 15). African Adults: Intramuscular injection of AMA1-DiCo/GLA-SE (*n* = 18) or placebo (*n* = 18). AMA1-DiCo (50 mg) was administered intramuscularly at baseline, Week 4 and 26.	The main immunogenic response noted was an increase in IgG. The AMA1-DiCo malaria vaccine with Alhydrogel^®^ group caused a 100-fold IgG increase from baseline and a 200–300-fold IgG increase when adjuvanted with GLA-SE. In African volunteers, immunization resulted in increased IgG levels that surpassed those of the European volunteers by 4-fold. Volunteers immunized also displayed a strong Th2 cell response that was present in more than 50% of the volunteers and detected by an IL-5 ELISPOT assay
2013	Pandemic influenza A (H1N1) 2009 vaccination in children: A UK perspective [19]	Adjuvanted vaccine AS03 for H1N1	6 months to 12 years old <10 years old 10–24 years old 30–60 years old	2 doses regimen of the Adjuvanted vaccine AS03 for children Review does not describe intervention for adults.	77% effectiveness in children <10 years old. 100% effectiveness in ages 10–24 years old. 89–92% in children (6 months to 12 years) and 69–89% in adults (30–60 years).
2011	Panvax^®^: a monovalent inactivated unadjuvanted vaccine against pandemic influenza A (H1N1) 2009 [20]	Panvax^®^ vaccine	Adults and children (6 months to 64 years old)	15-μg dose of Panvax^®^, a monovalent inactivated adjuvanted vaccine	Patients established > 90% seroprotection.
2011	A fast-tracked influenza virus vaccine produced in insect cells [21]	FluBlok vaccine	Study PSC01: Healthy adults aged 18–49 years old; enrolled during 2004–2005 flu season PSC04: Healthy adults aged 18–49 years old; enrolled during 2007–2008 flu season	Study PSC01: Subjects were vaccinated with either FluBlok 135 μg (*n* = 153) or 75 μg (*n* = 151), or a saline placebo (*n* = 154) Study PSC04: Subjects were randomized to receive FluBlok 135 μg (*n* = 2344) or placebo (*n* = 2304)	Protective efficacy was established against culture-confirmed CDC-ILI was 85.5% overall (95% CI 23.7, 98.5). This study also revealed statistically significant reduction in culture-confirmed CDC-ILI between subjects who received FluBlok (135 μg) vs. placebo (*p* = 0.0146).
2009	FluBlok, a next generation influenza vaccine manufactured in insect cells [22]	FluBlok vaccine	Study PSC01: Healthy adults aged 18–49 years old; enrolled during 2004–2005 flu season Study PSC03: Healthy adults age ≥ 65 years old; enrolled during 2006–2007 flu season Study PSC04: Healthy adults aged 18–49 years old; enrolled during 2007–2008 flu season Study PSC06: Healthy adults aged 50–64 years old; enrolled during 2007–2008 flu season	Study PSC01: Subjects were randomized to receive FluBlok 135 μg (*n* = 153) or 75 μg (*n* = 151), or placebo (*n* = 154) Study PSC03: Subjects were randomized to receive FluBlok 135 μg (*n* = 431) or trivalent, inactivated influenza virus vaccine (Fluzone^®^) (*n* = 430) Study PSC04: Subjects were randomized to receive FluBlok 135 μg (*n* = 2344) or placebo (*n* = 2304) Study PSC06: Subjects were randomized to receive FluBlok 135 μg (*n* = 299) or a trivalent, inactivated influenza virus vaccine (Fluzone^®^) (*n* = 302)	Study PSC01: FluBlok provided 87% seroprotection against A/H1N1 and 100% seroprotection against A/H3N2 (95% CI 98, 100) with the 135-μg dose. Study PSC03: FluBlok provided 95% seroprotection against A/H1N1 (95% CI 92, 97) and 97% seroprotection against A/H3N2 (95% CI 94, 98) Study PSC04: FluBlok provided 98% seroprotection against A/H1N1 (95% CI 97, 99) and 96% seroprotection against A/H3N2 (95% CI 94, 98). Study PSC06: Flublok provided 96% seroprotection against A/H1N1 (95% CI 94, 98) and 85% seroprotection against A/H3N2 (95% CI 81, 89).
2009	(1) The Enigma of the H1N1 Flu: Are You Ready? [23] (2) MMWR: Update on Influenza A (H1N1) 2009 Monovalent Vaccines [24]	H1N1 vaccine	Adults aged ≥ 18 years old	One 15-μg injection of the H1N1 vaccine	After 21 days, 97% of these adults had enough antibodies for optimal protection against the virus. Antibody titers of 1:40 or more (hemagglutination-inhibition assay) were observed in 116 (97%) of 120 adults who received the 15-μg dose.
2008	BiovaxID, a personalized therapeutic vaccine against B-cell lymphomas [25]	BiovaxID vaccine	Patients with follicular non-Hodgkin’s lymphoma in primary or secondary remission after chemotherapy treatment	Phase I Intervention: five subcutaneous immunizations of BiovaxID to 41 patients with follicular non-Hodgkin’s lymphoma Phase II Intervention: four monthly subcutaneous injections of BiovaxID with a sample size of 20 in complete remission after six or more monthly cycles of proMACE chemotherapy.	Phase 1 Result: 85% had antibody responses, 35% had cellular responses, and 87% of the patients who responded to the vaccine were able to remain tumor free. Phase I also revealed a progression-free period for vaccinated patients with a response of 7.9 years Phase II Result: Phase II of this trial yielded results showing 45% disease-free survival and a 95% overall survival among patients. BiovaxID treatment after 36 months showed approximately 100% improvement in median disease-free survival. (*p* = 0.024).
2003	Cancer Vaccine THERATOPE^®^—Biomira [26]	THERATOPE^®^	Patients with ovarian and breast cancer receiving HDC/ASCT	Five doses of THERATOPE^®^ after HDC/ASCT	Patients who did not receive THERATOPE^®^ had a 2-fold and 1.7-fold higher risk of death and relapse, respectively.
2012	The Failed Theratope Vaccine: 10 Years Later [27]	THERATOPE^®^	Patients with metastatic breast cancer receiving cyclophosphamide (*n* = 1030)	After initial dose, THERATOPE^®^ was given monthly for 4 months, and then quarterly until disease progression	Week-12 antibody titers showed high IgG in the treatment group and undetectable levels in the control group. Overall median survival between the treatment and control groups were not statistically significant (23.1 months vs. 22.3 months, respectively).
2006	Rotavirus Vaccine: Early Introduction in Latin America—Risks and Benefits [28]	Rotarix vaccine against attenuated serotype G1 rotavirus strain	Infants and young children	N/A	The efficacy of the vaccine against severe rotavirus gastroenteritis and associated hospitalization was 85% (*p* < 0.001vs. placebo) and even amounted to 100% against more severe cases of gastroenteritis. It also reduced the hospitalizations of diarrhea by 42% (95% confidence interval). The overall efficacy of Rotarix against the G1 rotavirus strain was seen to be 91%.

Abbreviations: AMA1-DiCo: Apical Membrane Antigen 1 Diversity Covering; CDC-ILI: Influenza-Like Illness specified by the CDC; GLA-SE: Glucopyranosyl Lipid Adjuvant-Stable Emulsion; HDC/ASCT: High-Dose Chemotherapy/Autologous Stem Cell Transplant; IgG: Immunoglobulin G; MMWR: Morbidity and Mortality Weekly Report; proMACE: Prolix (prednisone), methotrexate, Adriamycin (doxorubicin), cyclophosphamide, and etoposide.

**Table 3:** Articles reviewed for safety.

Publication Date	Study Name	Fast-Tracked Vaccine	Patient Population	Intervention	Results
2019	The Candidate Blood-stage Malaria Vaccine P27A Induces a Robust Humoral Response in a Fast Track to the Field Phase 1 Trial in Exposed and Nonexposed Volunteers [17]	P27A peptide vaccine against malaria	Phase 1a: 16 healthy European volunteers not previously exposed to malaria (8 volunteers per group) Phase 1b: 40 malaria exposed African volunteers (4 groups: 8 people getting vaccine, 2 people getting control)	P27A antigen (10 or 50 μg), adjuvanted with Alhydrogel or GLA-SE (2.5 or 5 μg), or control rabies vaccine (Verorab) were administered intramuscularly to 16 malaria-nonexposed and 40 malaria-exposed subjects on days 0, 28, and 56.	Phase 1a: Most common systemic adverse effects were tiredness (48.5%) and headache (29.2%). There were no significant abnormal vital signs or other severe effects. Phase 1b: 64.1% experienced fatigue and headache. There were no significant abnormal vital signs.
2017	Safety and immunogenicity of a recombinant *Plasmodium falciparum* AMA1-DiCo malaria vaccine adjuvanted with GLA-SE or Alhydrogel^®^ in European and African adults: A phase 1a/1b, randomized, double-blind multi-center trial [18]	Recombinant *Plasmodium falciparum* AMA1-DiCo malaria vaccine adjuvanted with GLA-SE or Alhydrogel^®^	Cohort A: 30 European adults Cohort B: 36 African adults	Cohort A: Participants received AMA1-DiCo with either Alhydrogel^®^ (*n* = 15) or GLA-SE (*n* = 15) Cohort B: Participants received either AMA1-DiCo/GLA-SE (*n* = 18) or placebo (*n* = 18)	Cohort A: Local reactions (injection site pain, limited arm abduction at the shoulder) were seen with 93% of the Alhydrogel^®^ group and 100% of the GLA-SE group. 1/30 participants experienced epilepsy unrelated to the vaccination. There were no further systemic reactions reported. Cohort B: Local reactions were experienced by 33% of the GLA-SE group and 17% of the placebo group. Systemic reactions were seen in 22% of the GLA-SE group and 22% of the placebo group.
2003	Cancer Vaccine THERATOPE^®^—Biomira [26]	THERAPTOPE^®^	30 patients with ovarian cancer	The 30 participants of the phase II study were randomized to receive either 10 or 100 units of THERATOPE^®^ administered subcutaneously at weeks 0, 2, and 5, and then every 4 weeks. Total of 6 doses.	The preliminary results in the R&D profile indicated that the vaccine was well-tolerated. All patients experienced mild flu-like syndrome for 2 to 5 days after vaccination. Only one patient discontinued treatment due to adverse events (dyspnea and hypoxia after the fourth dose).
2012	The failed Theratope vaccine: 10 years later [27]	THERAPTOPE^®^	women with metastatic breast cancer	N/A	Phase II trials reported minimal toxic effects, primarily mild injection-site reactions and flu-like symptoms.
2006	Rotavirus Vaccine: Early Introduction in Latin America—Risks and Benefits [28]	Rotarix and RotaShield vaccines	Infants and young children	N/A	Shortly after RotaShield became available in the US (October 1998), there were about 100 cases of intussusception associated with vaccine administration. In 1999, RotaShield was quickly withdrawn from the market. The preliminary clinical data on Rotarix was not sufficient to make conclusions about safety.
2006	Sipuleucel-T: APC 8015, APC-8015, Prostate Cancer Vaccine—Dendreon [29]	Sipuleucel-T	Men with advanced prostate cancer D9901: 127 men D9902A: 98 men	D9901: Patients were randomized to receive either Sipuleucel-T (*n* = 82) or placebo (*n* = 45) D9902A: Patients were randomized to receive Sipuleucel-T (*n* = 65) or placebo (*n* = 33)	For both D9901 and D9902A trials, Sipuleucel-T was well-tolerated among the vaccine recipients.
2009	Integrated data from 2 randomized, double-blind, placebo-controlled, phase 3 trials of active cellular immunotherapy with sipuleucel-T in advanced prostate cancer [30]	Sipuleucel-T	Men with advanced prostate cancer D9901: 127 men D9902A: 98 men	D9901: Patients were randomized to receive either Sipuleucel-T (*n* = 82) or placebo (*n* = 45) D9902A: Patients were randomized to receive Sipuleucel-T (*n* = 65) or placebo (*n* = 33)	Less than 3% of patients in D9901 and D9902A had treatment-related adverse events that prevented them from receiving all 3 infusions. Integrated data showed that the adverse reactions occurring at a higher rate (*p* ≤ 0.05) in the Sipuleucel-T group compared to the placebo group were chills, fever, headache, asthenia, dyspnea, vomiting and tremor. Additionally, these adverse reactions were primarily mild (grade 1 and 2, duration 1 to 2 days).
2010	Sipuleucel-T immunotherapy for castration-resistant prostate cancer [31]	Sipuleucel-T	512 men with metastatic castration-resistant prostate cancer	341 patients received Sipuleucel-T, and 171 patients received a placebo	Sipuleucel-T was generally well-tolerated in terms of adverse events and also prolonged survival in study patients
2014	Pandemic influenza A H1N1 vaccines and narcolepsy: vaccine safety surveillance in action [32]	H1N1-AS03-P vaccine	Adolescents in Finland, Sweden, Ireland, and the Netherlands, who received the vaccine	N/A	In 2010, reports of narcolepsy associated with vaccination in adolescents from Sweden and Finland prompted an investigation by the ECDC and VAESCO. The results of the investigation and other studies supported an increased risk of post-vaccination narcolepsy.
2011	Safety of pandemic H1N1 vaccines in children and adolescents [33]	Pandemic H1N1 vaccines (adjuvanted vs. non-adjuvanted)	Children and adolescents	N/A	Studies found that the adjuvanted vaccines were more reactogenic than non-adjuvanted vaccines. In terms of adverse events, they were both generally well-tolerated. Differing methodology between various studies made it difficult to make conclusions about the safety profile of these vaccines.

Abbreviations: ECDC: European Centre for Disease Prevention and Control; GLA-SE: Glucopyranosyl Lipid Adjuvant-Stable Emulsion; IgG: Immunoglobulin G; PSA: Prostate-Specific Antigen; R&D: Research and Development; VAESCO: Vaccine Adverse Event Surveillance and Communication Consortium.

**Table 4:** Articles reviewed for lessons learned.

Publication Date	Study Name	Fast-Tracked Vaccine	Patient Population	Lessons Learned
2013	Pandemic influenza A (H1N1) 2009 vaccination in children: A UK perspective [19]	Adjuvanted vaccine AS03 for H1N1	6 months to 12 years old <10 years old 10–24 years old 30–60 years old	The substantial developments in understanding influenza epidemiology, pandemic policy planning, and vaccinology. As well as the discovery that using vaccines with oil in water adjuvant systems results in great immunogenicity in the younger population
2011	Panvax^®^: a monovalent inactivated unadjuvanted vaccine against pandemic influenza A (H1N1) 2009 [20]	Panvax^®^ vaccine	Adults and children (6 months to 64 years old)	The future of pandemic vaccines lies in developing more broadly cross-protective preparations capable of preventing infection with both seasonal and pandemic strains if rapid containment of emerging viruses is to be achieved
2003	Cancer Vaccine THERATOPE^®^—Biomira [26]	THERAPTOPE^®^	95 patients with ovarian cancer in 12 different US sites	The vaccine did not advance past the checkpoints in clinical trials, so it was not put on the market.
2012	The failed Theratope vaccine: 10 years later [27]	THERAPTOPE^®^	1030 patients with metastatic breast cancer from 120 sites in 10 countries	THERAPTOPE^®^ did not meet pre-determined statistical endpoints in clinical trials, so it was not released to the market.
2006	Rotavirus Vaccine: Early Introduction in Latin America—Risks and Benefits [28]	Rotavirus vaccines	1000+ people in trials performed in 11 Latin American countries	Rotarix vaccine was safe and efficacious. Lessons learned from this study are that fast-tracking such vaccines would be of great importance to the safety of children in countries where deadly rotavirus is commonly found.
2003	HIV gp120 vaccine—VaxGen: AIDSVAX, AIDSVAX B/B, AIDSVAX B/E, HIV gp120 vaccine—Genentech, HIV gp120 vaccine AIDSVAX—VaxGen, HIV vaccine AIDSVAX—VaxGen [34]	AIDSVAX	5108 men who have sex with men and 309 at-risk women	When organizations collaborate and combine their resources, remarkable goals can be achieved.
2003	Understanding the Results of the AIDSVAX Trial|AVAC [35]	AIDSVAX	Patients at high-risk for HIV infection received vaccine (*n* = 3330) vs. placebo (*n* = 1679)	The trial provided important information on the logistics of conducting AIDS vaccine efficacy trials and development of future HIV/AIDS vaccines.
2009	Antiviral role of toll-like receptor-3 agonists against seasonal and avian influenza viruses [36]	Not a vaccine but was studied for H5N1	Mice	The study confirms the justification for regulatory agencies to consider fast-track development of drugs for prophylaxis and potentially the treatment of H5N1.
2015	Emergency treatment for exposure to Ebola virus: the need to fast-track promising vaccines [37]	VSVΔGZEBOV	One physician who had a needlestick in an Ebola treatment unit	It is important to have a sufficient supply of safe and effective vaccines that can be rapidly deployed in emergency situations.
2018	EBOVAC-Salone: Lessons learned from implementing an Ebola vaccine trial in an Ebola-affected country [38]	Ebola vaccine	Stage 1: 40 participants Stage 2: 730 participants	Research should be more closely incorporated into outbreak response planning, expediting timely and appropriate research projects.
2018	Ebola: Lessons on Vaccine Development [39]	Ebola vaccine	Many different species of animals such as lab mice, humanized mouse, hamster, guinea pig, ferret, and non-human primate.	Studying other EBOV antigens so that time, money, and better results can be achieved earlier rather than having extra expenses and reduced optimization. The epidemic demonstrated the lack of preparation and limitations in our public health response. Since then, better planning and preparation have been discussed and implicated.
2018	Ebola vaccines—Where are we? [40]	Ebola vaccine	N/A	Good coordination and collaboration during future epidemics are necessary when it comes to developing a vaccine safely and effectively. Encouraging the world health organization to improve current structures so that response and preparation will be improved when a future epidemic occurs.
2020	Post-marketing studies: can they provide a safety net for COVID-19 vaccines in the UK? [41]	COVID-19 vaccines	Adults in the UK	COVID-19 vaccines are quickly progressing through clinical development with fast-tracking, and post-marketing surveillance and observational studies can help bridge gaps in clinical trial data, especially in regard to safety.

Abbreviations: COVID-19: Coronavirus Disease 2019; EBOV: Ebola Virus; FDA: Food and Drug Administration; HIV: Human Immunodeficiency Virus; R&D: Research and Development; UK: United Kingdom; US: United States; VSVΔGZEBOV: recombinant vesicular stomatitis virus-based Ebola vaccine.

## Data Availability

Not applicable.
